# 

**Published:** 1865-06

**Authors:** 


### CONTENTS.

### ORIGINAL CONTRIBUTIONS.

ART. XVIII. Report of the Special Committee on Diseases of the Eye. By E. L. Holmes, M.D., of Chicago. Presented to the Illinois State Medical Society, ... 321

ART. XIX. Hypodermic Injections for the Obliteration of Varicose Veins. A Clinical Lecture. By E. Andrews, M.D., Professor of Surgery in Chicago Medical College, ... 327

ART. XX. Epidemic Erysipelatous Fever at Waverly. By R. E. McVey, M.D., of Waverly, Ill. Presented to the Illinois State Medical Society, ... 331

ART. XXI. Necrosis as an Effect of Erysipelas. By E. Andrews, Prof. of Surgery in Chicago Medical College, ... 338

ART. XXII. Patents for Surgical Instruments. By X. ... 340

### SELECTIONS.

Clinical Lectures on Scriveners' Palsy. Delivered at St. Thomas' Hospital. By Samuel Solly, Esq., F.R.S., Senior Surgeon to the Hospital, 342

Tubercular Peritonitis, or Scrofulous Inflammation of the Abdominal Viscera. By W. H. Triplett, M.D., Washington, D.C., ... 356

A New Mode of Treating Diseases of the Cavity of the Nose. By J. L. W. Thudichum, M.D., M.R.C.P., Lettsomian Professor of Medicine of the Medical Society of London, ... 362

Bacteridia and Malignant Pustule, ... 368

The Starvation of Prisoners, ... 371

### EDITORIAL.

Sanitary Commissions and Fairs. Their Moral and Medical Results, ... 376

Transactions of the State Medical Society, ... 378

The American Medical Association, ... 379

Chicago Academy of Sciences, ... 380

Soldier's Home, ... 380

The Committee on Ophthalmology, (Illinois State Medical Society,) ... 381

Personal.—Dr. H. A. Johnson.—Prof. Chas. Pope, of St. Louis, ... 381

Compliment to an Army Surgeon, ... 381

Parisian Hospitals, ... 381

